# Perioperative analgesic effects of opiranserin in canine castration: a pilot study

**DOI:** 10.3389/fvets.2026.1954078

**Published:** 2026-10-05

**Authors:** Seongwon Lee, Seonbin Oh, Inhyung Lee, Won-Gyun Son

**Affiliations:** Department of Veterinary Clinical Sciences, College of Veterinary Medicine, Seoul National University, Seoul, Republic of Korea

**Keywords:** opiranserin, acute pain management, 5-HT2A antagonist, glycine transporter 2 inhibitor, intraoperative nociception, postoperative pain, canine castration, rescue analgesia

## Abstract

**Introduction:**

Opiranserin, a non-opioid analgesic acting as a glycine transporter 2 inhibitor and 5-HT2A receptor antagonist, was evaluated for reducing intraoperative rescue analgesia and postoperative pain in dogs undergoing castration under isoflurane anesthesia.

**Materials and methods:**

Eight healthy male Beagle dogs were randomized to receive a continuous rate infusion of opiranserin (5 mg·kg^−1^·h^−1^ for 1 h before induction, then 0.33 mg·kg^−1^·h^−1^ for 9 h; *n* = 4) or 0.9% saline (*n* = 4). Anesthesia was induced with alfaxalone and maintained with isoflurane (1.5% end-tidal); all dogs received meloxicam and cefazolin preoperatively, without locoregional analgesia. Hemodynamic responses (ΔMAP, ΔHR) were recorded across 11 surgical events; rescue remifentanil was given when both increased ≥20% from baseline. Postoperative pain was scored with the Glasgow Composite Measure Pain Scale–Short Form (GCMPS-SF) from 30 min after extubation (T0) through T7, with rescue analgesia at scores ≥5. Primary outcomes were intraoperative rescue analgesia frequency and postoperative GCMPS-SF scores; secondary outcomes were intraoperative hemodynamic responses, postoperative rescue consumption, and adverse effects. Data were analyzed with Mann–Whitney *U* tests and linear mixed models with Bonferroni-adjusted comparisons.

**Results:**

The opiranserin group required fewer intraoperative rescue interventions than the normal saline group (median 2 [2–2] vs. 6.5 [3–9]; *p* = 0.021). GCMPS-SF scores showed significant effects of group (*p* = 0.030), time (*p* = 0.047), and group × time interaction (*p* = 0.034), with lower scores in the opiranserin group at T1 (adjusted *p* = 0.012) but not at other time points. Among secondary outcomes, significant overall effects of group were found for ΔMAP (*p* = 0.011) and ΔHR (*p* = 0.048), and of surgical event for both (*p* < 0.001 and *p* = 0.008), with no group × event interaction; no individual event differed significantly after correction. Cumulative postoperative rescue analgesic consumption and the incidence of adverse effects did not differ between groups.

**Conclusion:**

These preliminary findings suggest that opiranserin may reduce intraoperative rescue analgesia requirements and attenuate postoperative pain in dogs, warranting confirmation in larger trials. The absence of significant adverse effects is reassuring but should be interpreted cautiously given the small sample size.

## Introduction

Effective perioperative analgesia is essential in veterinary patients because inadequate pain control can increase sympathetic and neuroendocrine stress responses and contribute to central sensitization (1, 2). Current guidelines recommend multimodal analgesia using opioids, non-steroidal anti-inflammatory drugs (NSAIDs), and local anesthetics as first-tier agents for acute surgical pain in dogs and cats (3). Among these, opioids have traditionally been considered the cornerstone of perioperative pain management, owing to their reliable and dose-dependent analgesic efficacy (4). Although opioids provide potent analgesia, their use may be limited by adverse effects including bradycardia, respiratory depression, dysphoria, vomiting, nausea, and salivation (4). These limitations have increased interest in non-opioid analgesics that can reduce perioperative nociception while minimizing opioid-related adverse effects.

Opiranserin is a novel non-opioid analgesic that inhibits glycine transporter 2, thereby enhancing spinal glycinergic inhibition, and antagonizes the 5-hydroxytryptamine 2A receptor, thereby attenuating descending serotonergic pain facilitation (5–7). Several human clinical studies have examined the postoperative analgesic efficacy of opiranserin (8, 9). In a phase 2 trial involving 59 patients undergoing laparoscopic gastrectomy, opiranserin significantly reduced postoperative pain intensity and decreased opioid consumption by 29.5% compared with placebo (8). In a subsequent phase 3 trial involving 284 patients undergoing laparoscopic colectomy, opiranserin reduced patient-controlled analgesia requests by 60.2% and opioid consumption by 30.8% (9). In veterinary medicine, however, only one study has evaluated its analgesic properties, demonstrating increased mechanical nociceptive thresholds in six Beagle dogs compared with both normal saline (negative control) and low-dose remifentanil (4 μg·kg^−1^·h^−1^, positive control), with opiranserin exceeding remifentanil from 60 to 240 min of infusion (10). Thus, existing evidence remains confined to human postoperative clinical trials and a single experimental veterinary nociception model; opiranserin's perioperative analgesic efficacy in an actual clinical surgical setting has not yet been evaluated.

Safety data have also been reported for opiranserin. In the phase 1 single ascending dose study (single 4-h infusion of up to 8 mg·kg^−1^), drug-related adverse events occurred in 13 of 46 healthy volunteers (28.3%) and comprised somnolence, nausea, dizziness, and headache; all were mild and transient, occurred predominantly at the highest doses (6–8 mg·kg^−1^), and resolved after discontinuation (5). In the phase 2 and phase 3 trials, the incidence of treatment-emergent adverse events was comparable between the opiranserin and placebo groups, and events considered drug-related were infrequent and mild to moderate in severity, resolving without intervention (8, 9). No serious adverse events attributed to opiranserin were reported in any trial. In the single experimental study conducted in Beagle dogs, the principal adverse effects of opiranserin were vomiting in two of six dogs and nausea-like signs in five of six dogs; all adverse effects resolved within 30 min of discontinuing the infusion, and no sedation, psychomimetic signs, or neurological signs were observed (10). Taken together, the available efficacy and safety data support further evaluation of opiranserin, but neither has been assessed in dogs undergoing surgery.

Accordingly, the present study evaluated the perioperative analgesic efficacy of opiranserin in male dogs undergoing castration, one of the most commonly performed elective procedures in small animal practice (11). This model was selected because the nociceptive events are easy to detect and their intensity is minimally influenced by the surgeon, providing a standardized and reproducible surgical stimulus of mild-to-moderate anticipated postoperative pain with well-defined intraoperative nociceptive events that enable systematic hemodynamic monitoring (12). Within this framework, the primary outcomes were intraoperative rescue analgesia frequency and postoperative Glasgow Composite Measure Pain Scale–Short Form (GCMPS-SF) scores, chosen to capture analgesic efficacy during the intraoperative and postoperative phases, respectively. Secondary outcomes were changes in mean arterial pressure (ΔMAP) and heart rate (ΔHR) across 11 standardized surgical events, cumulative postoperative rescue analgesic consumption, and the incidence of adverse effects.

Given the absence of prior clinical data, this pilot study was designed as an exploratory investigation to generate preliminary estimates of opiranserin's perioperative analgesic effects in a clinical surgical setting. We hypothesized that continuous rate infusion (CRI) of opiranserin would reduce intraoperative rescue analgesia requirements and postoperative GCMPS-SF scores, attenuate hemodynamic responses to surgical stimulation, and decrease postoperative rescue analgesic consumption compared with normal saline.

## Materials and methods

### Ethical approval

All experimental procedures were approved by the Institutional Animal Care and Use Committee of Seoul National University (IACUC No. SNU-250728-4-2). Animals were housed and cared for in accordance with the guidelines of the Korean Laboratory Animal Act.

### Animals and study design

Eight healthy, sexually intact male Beagle dogs were enrolled in this randomized, blinded, parallel-group controlled pilot study. In accordance with the pilot study design, four dogs were allocated to each group: a normal saline (N/S) group (*n* = 4) and an opiranserin group (*n* = 4). All dogs underwent castration under general anesthesia.

Group allocation was determined by simple randomization using a random number table, performed by an independent third party not involved in the study. The allocation sequence was concealed from the surgeon, surgical assistant, anesthesiologist, and pain assessor throughout the study. All surgeries were performed by the same experienced veterinary surgeon, who had performed more than 50 canine castrations before the study, using a standardized open scrotal orchiectomy technique with a separate incision over each testis.

### Sample size

Because no previous surgical study in dogs provided reliable estimates of the effect size and variability for the selected outcomes, a formal *a priori* sample size calculation was not performed. Eight dogs, with four allocated to each group, were included in this exploratory pilot study to obtain preliminary estimates of treatment effects and variability while limiting animal use. Accordingly, the results were interpreted as preliminary and hypothesis-generating.

### Drug administration

Opiranserin was diluted in normal saline and administered as a CRI beginning 1 h before anesthetic induction at a loading dose of 5 mg·kg^−1^·h^−1^ for 1 h, followed by a maintenance dose of 0.33 mg·kg^−1^·h^−1^ for 9 h. The N/S group received 0.9% saline at an equivalent volume and rate. Drug preparation was performed by a dedicated investigator blinded to group allocation.

### Dose selection and pharmacokinetic rationale

The opiranserin dosing regimen was established using an exposure-based, model-informed approach informed by human clinical exposure data and canine pharmacokinetic simulations. In humans, combined plasma concentrations of opiranserin and its active metabolite VVZ-368 associated with analgesic efficacy have been reported at approximately 2,000–2,500 ng·ml^−1^. Because systemic-to-CNS exposure relationships differ across species and canine CNS exposure was expected to be greater relative to systemic plasma concentration, a lower provisional systemic target of approximately 1,000–1,250 ng·ml^−1^ was selected for the initial infusion phase. This value was treated as a pragmatic design target rather than a validated canine therapeutic concentration. The regimen was designed as a two-phase infusion, with a loading phase intended to rapidly achieve pharmacologically relevant exposure before surgical stimulation and a lower maintenance phase intended to sustain exposure while limiting prolonged systemic accumulation. Monte Carlo pharmacokinetic simulations (100 virtual subjects), based on the combined exposure of opiranserin and VVZ-368, supported an initial infusion of 5 mg·kg^−1^·h^−1^ for 1 h followed by 0.33 mg·kg^−1^·h^−1^ for 9 h, corresponding to a total dose of approximately 8 mg·kg^−1^ over 10 hours.

### Anesthetic protocol

Anesthesia was induced with alfaxalone (2 mg·kg^−1^ IV) and maintained with isoflurane at a fixed end-tidal concentration of 1.5%; all dogs breathed spontaneously throughout the procedure. The concentration was held constant so that hemodynamic responses to surgical events would reflect differences in analgesic efficacy rather than variation in anesthetic depth. Anesthetic depth was monitored continuously by jaw tone, palpebral reflex, eye position, and spontaneous movement. Isoflurane was to be increased in 0.1% increments if signs of insufficient depth were observed.

No opioid premedication or locoregional analgesia was administered, in order to isolate the analgesic contribution of opiranserin from that of other agents. All dogs received meloxicam as a baseline analgesic, and standardized intraoperative and postoperative rescue analgesia was provided to any dog meeting the predefined criteria.

### Perioperative management

Meloxicam (0.2 mg·kg^−1^ IV) was administered immediately after induction as a perioperative analgesic (13), and cefazolin (22 mg·kg^−1^ IV) was administered 30 min prior to skin incision as antimicrobial prophylaxis in accordance with the institutional protocol in place at the time of the study. Fluid therapy was administered using Lactated Ringer's solution at 10 mL·kg^−1^·h^−1^ for 1 h prior to induction, then reduced to 5 mL·kg^−1^·h^−1^ from induction through the end of surgery.

The protocol specified the following interventions for intraoperative complications: ephedrine (0.05 mg·kg^−1^ IV) for hypotension (MAP < 60 mmHg); glycopyrrolate (5 μg·kg^−1^ IV) for bradycardia accompanied by hypotension (HR < 60 beats/min with concurrent MAP < 60 mmHg); and active warming for hypothermia (rectal temperature < 36.5 °C). Body temperature was monitored continuously via rectal probe throughout anesthesia.

### Intraoperative nociception assessment

Hemodynamic variables including MAP and HR were continuously monitored via an arterial catheter placed in the right dorsal pedal artery and recorded automatically using a vital-sign recording program (Vital Recorder^®^). 11 standardized surgical events were manually recorded: E1, skin incision of the first side; E2, exteriorization of the first testis; E3, tearing of the ligament of the tail of the epididymis of the first testis; E4, clamping of the spermatic cord of the first testis; E5, transection of the spermatic cord of the first testis; E6, skin incision of the second side; E7, exteriorization of the second testis; E8, tearing of the ligament of the tail of the epididymis of the second testis; E9, clamping of the spermatic cord of the second testis; E10, transection of the spermatic cord of the second testis; and E11, wound closure (14).

For each surgical event, the mean value of MAP and HR recorded during the 10 s immediately preceding the event was designated as the pre-event baseline. The maximum values of each variable from the time of the event until the onset of the next event were identified, and the absolute change from baseline (ΔMAP and ΔHR, expressed as absolute change in mmHg and beats/min, respectively) was calculated.

### Postoperative pain and adverse effects assessment

Postoperative pain was assessed using the GCMPS-SF, a validated multidimensional pain assessment tool incorporating vocalization, attention to the painful area, mobility, response to touch, demeanor, and posture/activity (15). All assessments were performed by a single trained investigator who was blinded to group allocation throughout the study. Because dogs were individually housed in standard cages during the assessment period, and because interrupting the study CRI to permit ambulation would have altered exposure to the investigational treatment and confounded the results, the mobility subscale was omitted, reducing the maximum possible score from 24 to 20 points (15).

Pain assessments were performed at 30 min post-extubation (T0) and hourly from T1 to T7, for a total of eight evaluations. The CRI was discontinued within 1 h after T7, and no further pain assessments were conducted thereafter. All dogs were fully conscious and capable of spontaneous movement at T0, confirming adequate recovery from anesthesia prior to the commencement of pain scoring. Sedation was not formally scored in this study. The occurrence of adverse effects, including vomiting, nausea, and lip-smacking, was recorded throughout the postoperative observation period (10).

For each dog, the cumulative dose of remifentanil administered as postoperative rescue analgesia (expressed in μg·kg^−1^) was recorded as the measure of postoperative rescue analgesic consumption.

### Rescue analgesia

A simultaneous increase of ≥20% in both MAP and HR from the pre-event baseline was defined as the criterion for administering intraoperative rescue analgesia (16). Remifentanil (0.5 μg·kg^−1^ IV) was used as the rescue agent (17). The timing and total frequency of rescue analgesia were recorded. Postoperative rescue analgesia consisted of remifentanil administered as a CRI (4 μg·kg^−1^·h^−1^) for 1 h, initiated when the GCMPS-SF score reached ≥5 at any scheduled assessment time point (15, 18). If the score remained ≥5 at the subsequent hourly assessment, the CRI was maintained for an additional hour; if the score fell below 5, the CRI was discontinued. Remifentanil was selected as the postoperative rescue agent because its ultrashort duration of action (3–10 min) and rapid offset allow return to baseline analgesic status within each hourly assessment interval, thereby minimizing carryover effects on subsequent GCMPS-SF scoring (19).

### Statistical analysis

The primary outcomes — intraoperative rescue analgesia frequency and postoperative GCMPS-SF pain scores — were analyzed as follows. Intraoperative rescue analgesia frequency was compared between groups using the Mann–Whitney *U*-test, with the corresponding Hodges–Lehmann estimate of the median difference and its 95% confidence interval reported as the effect size. Postoperative GCMPS-SF scores were analyzed using a linear mixed model (LMM) with group, time (T0–T7), and their interaction as fixed effects and individual dog as a random intercept. Fixed effects were evaluated by likelihood ratio tests (χ^2^) on models fitted by maximum likelihood estimation. Estimated marginal means and per-timepoint between-group contrasts were derived from the model fitted by restricted maximum likelihood estimation, with degrees of freedom obtained by the Kenward–Roger approximation, and *p*-values adjusted using the Bonferroni correction across the eight time points.

The secondary outcomes — intraoperative hemodynamic responses, cumulative postoperative rescue analgesic consumption, and adverse effect incidence — were analyzed as follows. Intraoperative hemodynamic data were analyzed using an LMM with group (N/S group vs. opiranserin group), surgical event (E1–E11), and their interaction as fixed effects and individual dog as a random intercept, fitted using restricted maximum likelihood estimation; between-group differences at each surgical event were assessed using the estimated fixed effect coefficients (β) and associated *t*-tests with df = 6, with *p*-values adjusted using the Bonferroni correction across 11 surgical events. Cumulative postoperative rescue analgesic consumption and adverse effect incidence were compared between groups using the Mann–Whitney *U*-test. A *p*-value of < 0.05 was considered statistically significant.

Prior to fitting each LMM, the normality of residuals was assessed using the Shapiro–Wilk test, and homoscedasticity was evaluated by visual inspection of residual plots. The random intercept structure was selected as the most parsimonious model based on likelihood ratio testing. Confidence intervals are presented unadjusted for multiple comparisons and describe the precision of each individual estimate, whereas *p*-values are Bonferroni-adjusted across comparisons where indicated; consequently, an interval excluding the null value does not necessarily correspond to a significant adjusted *p*-value. Given the exploratory pilot design and the multiplicity of outcomes analyzed, all statistically significant findings should be interpreted with caution, as the risk of Type I error is inherently elevated in an underpowered study with numerous endpoints.

## Results

### Primary outcomes

The mean surgical time was 26.5 ± 8.5 min (range, 18–40 min). The opiranserin group required significantly fewer intraoperative rescue analgesia interventions than the N/S group [median 2 (range 2–2; all four dogs received rescue at exactly two events) vs. 6.5 (range 3–9); Hodges–Lehmann median difference 4.0 interventions, 95% CI 1.0–7.0; Mann–Whitney *U*-test, *p* = 0.021]. Across the 11 standardized surgical events, all four dogs in the opiranserin group received rescue analgesia at exactly two events each, although these rescue events occurred at different surgical steps among individual dogs. In contrast, N/S group dogs received rescue analgesia between three and nine times, with every individual in the N/S group exceeding the rescue count of any dog in the opiranserin group (Figure 1). Notably, at E2 (exteriorization of the first testis) — the most nociceptive event in this surgical model — all four dogs in the N/S group required at least one rescue intervention, whereas only one dog in the opiranserin group required rescue at this event.

**Figure 1:**
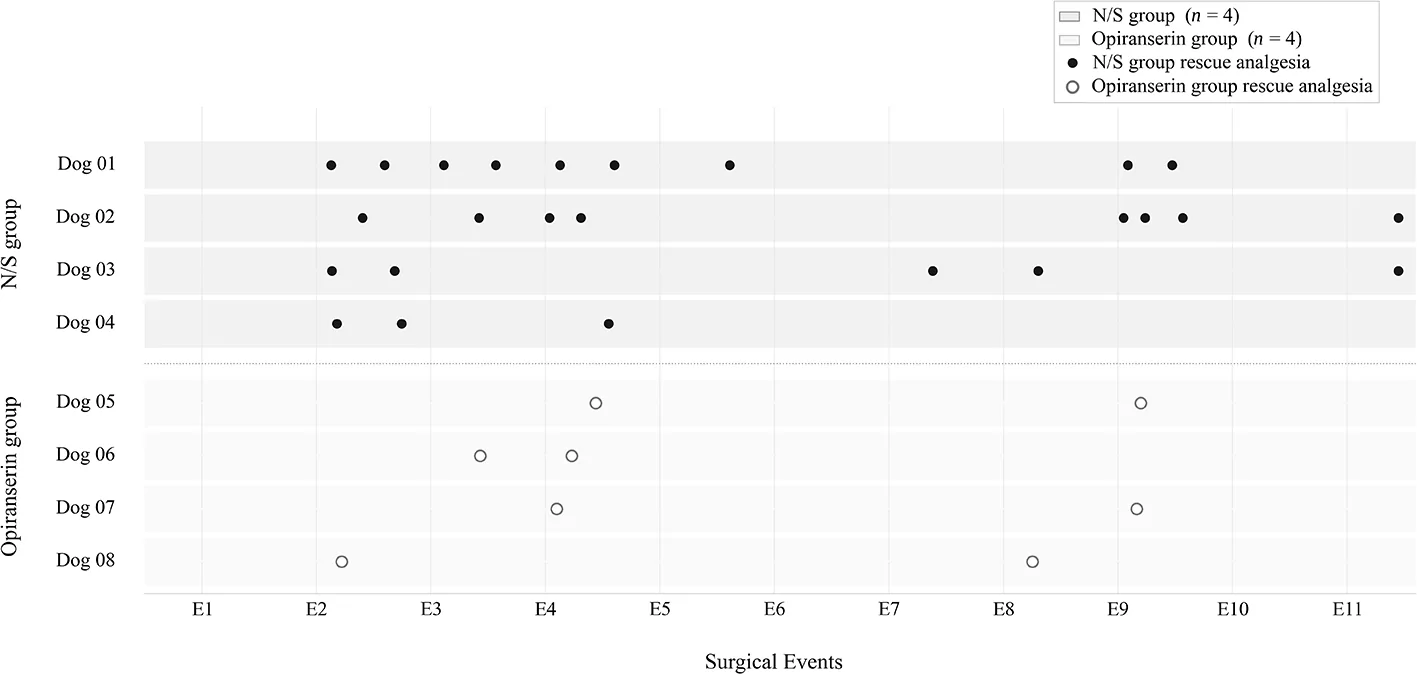
Intraoperative rescue analgesia frequencies per individual subject across surgical events. Filled circles (•) and open circles (°) indicate rescue events in the N/S group (*n* = 4) and opiranserin group (*n* = 4), respectively. The dotted line separates the two groups. E1, skin incision of the 1st side; E2, exteriorization of the 1st testis; E3, tearing of the ligament of the tail of the epididymis of the 1st testis; E4, clamping of the spermatic cord of the 1st testis; E5, transection of the spermatic cord of the 1st testis; E6, skin incision of the 2nd side; E7, exteriorization of the 2nd testis; E8, tearing of the ligament of the tail of the epididymis of the 2nd testis; E9, clamping of the spermatic cord of the 2nd testis; E10, transection of the spermatic cord of the 2nd testis; E11, wound closure.

Postoperative GCMPS-SF scores are summarized in Figure 2. LMM analysis revealed significant overall group (χ^2^(1) = 4.72, *p* = 0.030), time (χ^2^(7) = 14.23, *p* = 0.047), and group × time interaction (χ^2^(7) = 15.19, *p* = 0.034) effects. Per-timepoint comparisons with Bonferroni correction across the eight time points identified a significant between-group difference at T1 (the first hourly postoperative assessment, 1.5 h post-extubation), where the opiranserin group showed lower GCMPS-SF scores than the N/S group (median 1.0 [range 1–1] vs. 3.5 [range 1–6]; estimated mean difference 2.50, 95% CI 1.10–3.90; Bonferroni-adjusted *p* = 0.012). No significant between-group differences were observed at the remaining time points (T0, T2–T7; all adjusted *p* > 0.05).

**Figure 2:**
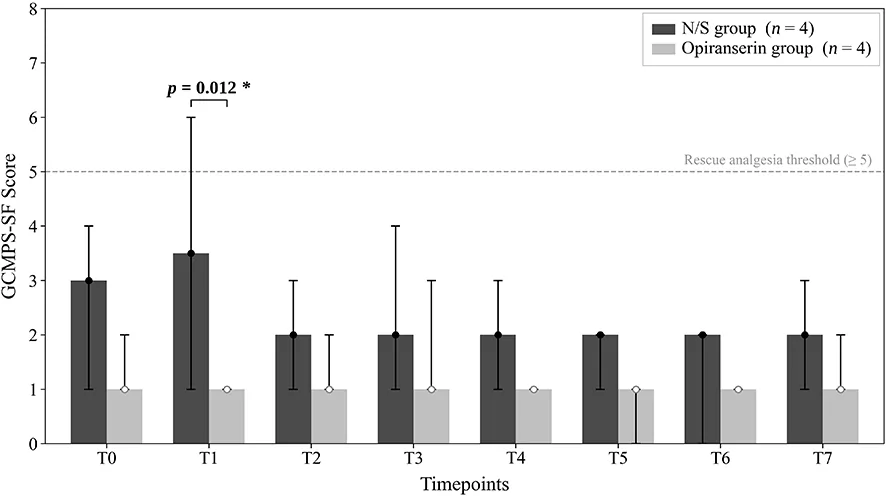
Postoperative Glasgow Composite Measure Pain Scale–Short Form (GCMPS-SF) scores over time in the N/S group (*n* = 4, dark bars, filled circles) and opiranserin group (*n* = 4, light bars, open circles). Data are presented as median (range); bar height and the superimposed circle indicate the median, and error bars indicate the range. The dashed horizontal line indicates the rescue analgesia threshold (score ≥ 5). T0, 30 min after extubation; T1, 1 h after T0; T2, 2 h after T0; T3, 3 h after T0; T4, 4 h after T0; T5, 5 h after T0; T6, 6 h after T0; T7, 7 h after T0. An asterisk (*) indicates a statistically significant between-group difference after Bonferroni correction (adjusted *p* < 0.05).

### Secondary outcomes

Linear mixed model analysis of ΔMAP and ΔHR across the 11 surgical events revealed significant overall effects of group [ΔMAP: χ^2^(1) = 6.44, *p* = 0.011; ΔHR: χ^2^(1) = 3.91, *p* = 0.048] and surgical event [ΔMAP: χ^2^(10) = 56.83, *p* < 0.001; ΔHR: χ^2^(10) = 23.89, *p* = 0.008], indicating that hemodynamic responses varied both between groups and across surgical events. No significant group × event interaction was detected [ΔMAP: χ^2^(10) = 18.19, *p* = 0.052; ΔHR: χ^2^(10) = 12.71, *p* = 0.241], suggesting that the pattern of hemodynamic change across events did not significantly differ between groups. Numerically, the most pronounced between-group differences were observed at E2 (exteriorization of the first testis), which represented the most nociceptive intraoperative stimulus in this surgical model. At E2, the N/S group exhibited a mean MAP increase of 36.7 ± 17.5 mmHg from baseline, whereas the opiranserin group showed a mean increase of 12.4 ± 5.0 mmHg (estimated mean difference (β) 24.4 mmHg, unadjusted 95% CI 9.2–39.5 mmHg; Bonferroni-adjusted *p* = 0.085). Similarly, HR increased by a mean of 35.6 ± 21.9 beats/min in the N/S group compared with 8.6 ± 6.1 beats/min in the opiranserin group (estimated mean difference (β) 27.0 beats/min, unadjusted 95% CI 5.9–48.1 beats/min; Bonferroni-adjusted *p* = 0.225). These differences did not reach statistical significance after Bonferroni correction and should be interpreted as numerical trends only, consistent with the exploratory nature of this pilot study (Figure 3).

**Figure 3:**
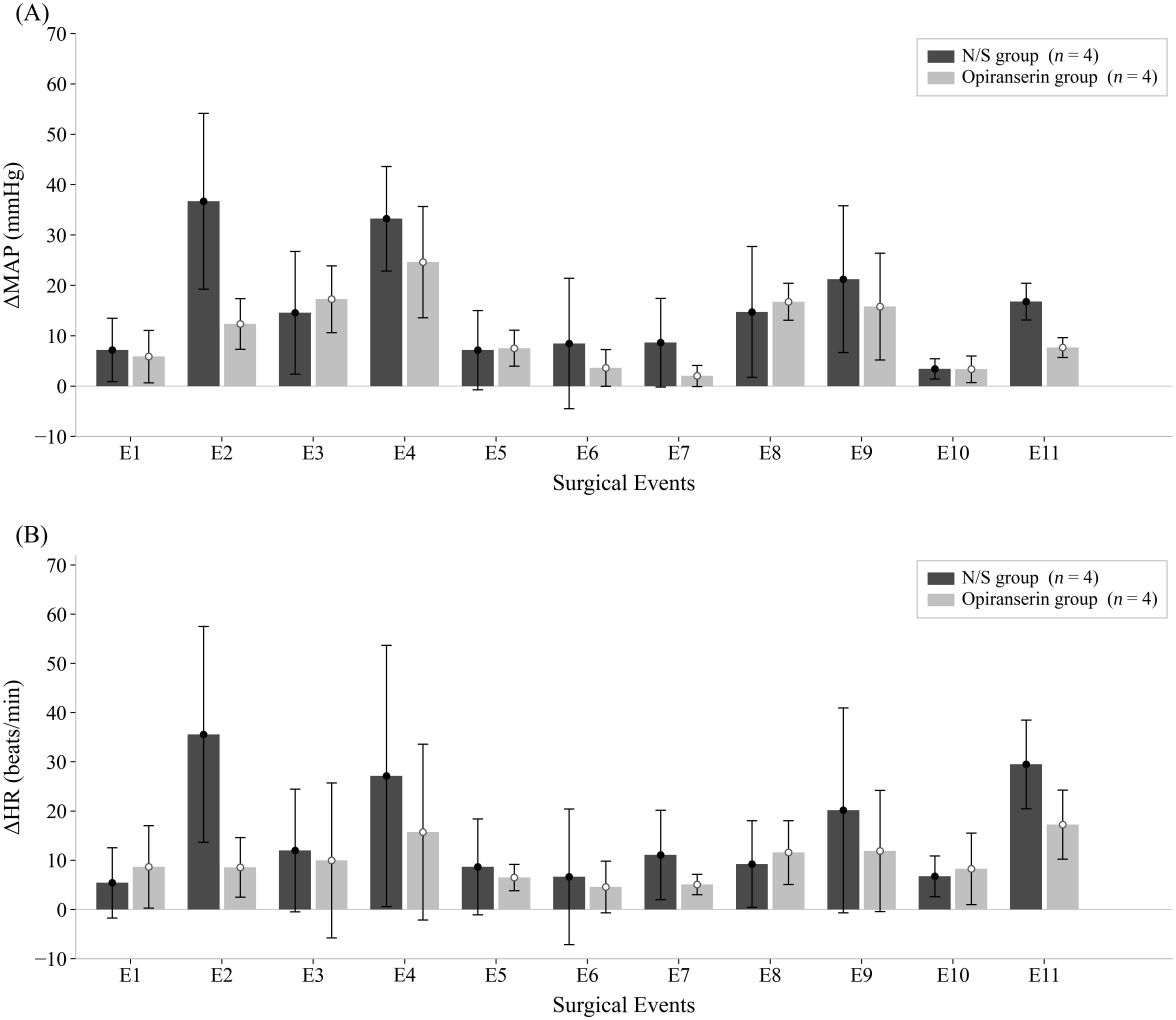
Intraoperative changes in mean arterial pressure (ΔMAP, **A**) and heart rate (ΔHR, **B**) at 11 predefined surgical events in dogs undergoing orchiectomy and receiving opiranserin (opiranserin group, *n* = 4) or normal saline (N/S group, *n* = 4) as perioperative analgesia. Data are expressed as mean ± SD. E1, skin incision of the 1st side; E2, exteriorization of the 1st testis; E3, tearing of the ligament of the tail of the epididymis of the 1st testis; E4, clamping of the spermatic cord of the 1st testis; E5, transection of the spermatic cord of the 1st testis; E6, skin incision of the 2nd side; E7, exteriorization of the 2nd testis; E8, tearing of the ligament of the tail of the epididymis of the 2nd testis; E9, clamping of the spermatic cord of the 2nd testis; E10, transection of the spermatic cord of the 2nd testis; E11, wound closure.

One dog in the N/S group required a single postoperative rescue analgesia intervention, receiving remifentanil CRI for 1 hour from T1 to T2 (total dose: 4 μg·kg^−1^), whereas no dog in the opiranserin group met the rescue threshold. No significant difference in cumulative postoperative rescue analgesic consumption was detected between groups (*p* = 0.453). Regarding adverse effects, one dog in the N/S group experienced one episode each of vomiting and nausea, and one other N/S dog exhibited lip-smacking once. In the opiranserin group, one dog showed one episode each of vomiting and nausea. Adverse effect incidence did not differ significantly between groups (all *p* > 0.05).

### Intraoperative anesthetic management

Anesthetic depth remained adequate throughout in all dogs, and no dog required an increase in the end-tidal isoflurane concentration.

Intraoperative hypotension before skin incision was managed with ephedrine in both groups. In the opiranserin group, three of four dogs received ephedrine (0.05 mg·kg^−1^ IV) on three occasions and one dog on two occasions, with a comparable number of doses administered in the N/S group. No dog in either group received glycopyrrolate, and no dog required active warming beyond the standard intraoperative warming measures.

## Discussion

The findings of this pilot study suggest that opiranserin may provide perioperative analgesic benefit in dogs undergoing castration. Dogs receiving opiranserin required fewer intraoperative rescue analgesic interventions, and LMM analyses indicated lower overall hemodynamic responses across surgical events. However, no event-specific between-group difference remained significant after adjustment for multiple comparisons. Postoperative GCMPS-SF score analyses also suggested a potential analgesic benefit in the early recovery phase, although the small sample size, low overall pain scores, and limited number of rescue events warrant cautious interpretation.

Intraoperative rescue analgesia was selected as a primary outcome because it represents a discrete, clinically actionable event — the point at which analgesic supplementation was deemed necessary — providing a direct and interpretable measure of intraoperative analgesic adequacy. The requirement for concurrent increases in both MAP and HR was adopted to enhance the specificity of the rescue criterion, restricting rescue administration to hemodynamic changes consistent with a nociceptive response rather than isolated autonomic fluctuations. Although this may reduce sensitivity compared with single-variable criteria, it was intended to minimize the number of rescue interventions and thereby preserve the discriminatory power of intraoperative rescue analgesia frequency — the primary outcome — between groups. Postoperative GCMPS-SF scores were selected as a co-primary outcome because the GCMPS-SF is a validated, multidimensional pain assessment tool specifically developed and standardized for use in dogs, incorporating behavioral and physiological indicators that collectively capture the clinical burden of postoperative pain (15). Together, these two outcomes were intended to provide complementary coverage of the perioperative analgesic profile of opiranserin, spanning both the intraoperative and postoperative phases of pain management.

The opiranserin group required fewer intraoperative rescue analgesia interventions than the N/S group, suggesting that opiranserin may provide intraoperative antinociception when administered as a CRI. Every dog in the N/S group required more rescue interventions than any dog in the opiranserin group, and this separation was most evident at E2 (exteriorization of the first testis) — the most nociceptive event in this surgical model — where all four N/S dogs required rescue compared with only one opiranserin dog. The rescue requirement was not only lower but also less variable in the opiranserin group, in which each dog required rescue at exactly two events, whereas the N/S group ranged from three to nine. However, with only four dogs per group, these observations cannot be interpreted with confidence and warrant confirmation in larger studies. In the postoperative period, a significant between-group difference in GCMPS-SF scores was observed only at T1 (Bonferroni-adjusted *p* = 0.012), with the opiranserin group showing lower pain scores. The absence of significant differences at the remaining time points may reflect the relatively mild postoperative pain associated with castration, which produced low scores in both groups and thus limited the scope for detecting a between-group difference (20).

Regarding the secondary outcomes, LMM analysis revealed significant overall effects of both group and surgical event on hemodynamic responses, with no significant group × event interaction, indicating that while hemodynamic responses varied across surgical events in both groups, opiranserin broadly attenuated the overall hemodynamic nociceptive response. Postoperative rescue analgesia was required in only one N/S dog and in none of the opiranserin dogs; although the direction of this difference is consistent with the intraoperative findings, it did not reach statistical significance (*p* = 0.453) and should be interpreted cautiously, as most dogs in both groups did not meet the rescue threshold. The absence of significant between-group differences in adverse effects is preliminary and cannot be used to draw definitive safety conclusions given the very small sample size; dedicated safety and tolerability studies in dogs are required.

The requirement for ephedrine to manage pre-incision hypotension was comparable between the opiranserin and N/S groups, indicating that opiranserin did not appreciably influence the incidence of anesthetic-related hypotension. Because ephedrine has an ultrashort duration of action and its final administration preceded skin incision by at least 5 min, with no further doses thereafter, its transient pressor effect had largely dissipated before the first surgical event and therefore did not confound the event-based ΔMAP and ΔHR analyses. No dog in either group required glycopyrrolate or active warming, further suggesting that opiranserin did not adversely affect intraoperative cardiovascular or thermoregulatory stability.

Opiranserin's dual mechanism — GlyT2 inhibition and 5-HT2A antagonism — enhances spinal glycinergic inhibition and attenuates descending serotonergic pain facilitation at the dorsal horn, thereby suppressing nociceptive transmission (21–24). Because this analgesic action is mediated at the spinal dorsal horn rather than through supraspinal sedative pathways, opiranserin has no known sedative properties, a potential advantage in the perioperative setting. The present pilot study represents the first clinical investigation of opiranserin in a companion animal species. Preliminary evidence of antinociceptive activity of opiranserin in dogs has been reported by Kim et al. (10), who demonstrated sustained increases in mechanical nociceptive thresholds throughout a 4-h infusion at 5 mg·kg^−1^·h^−1^ (total cumulative dose: 20 mg·kg^−1^), with antinociceptive effects exceeding those of low-dose remifentanil from 60 min onward. The present study extends these findings to a surgical setting, in which opiranserin showed preliminary evidence of perioperative analgesic activity at a substantially lower total cumulative dose of 8 mg·kg^−1^, administered as a loading infusion of 5 mg·kg^−1^·h^−1^ for 1 h followed by a maintenance infusion of 0.33 mg·kg^−1^·h^−1^ for 9 h. Notably, the incidence of gastrointestinal signs was numerically lower than in the preceding canine study: whereas Kim et al. reported vomiting in 2 of 6 dogs and nausea-like signs in 5 of 6 dogs, only one of four dogs in the present study exhibited either sign (10), although the differing group sizes preclude direct comparison. However, as nausea and vomiting were also observed in one dog in the normal saline group, a contribution from perioperative factors — including the effects of inhalant anesthetic agents, a recognized trigger of emesis in dogs, as well as postoperative pain mediated through shared neurokinin-1 and substance P signaling pathways — cannot be excluded (25, 26). Whether the gastrointestinal signs observed in the opiranserin group represent a true drug-related adverse effect or an incidental perioperative event therefore remains uncertain.

Beyond its lower total dose, the temporal profile of this regimen was deliberately shaped to concentrate drug exposure over the perioperative period. The high initial infusion rate was selected to reach the target rapidly; the simulated mean combined plasma concentration rose to approximately 1,307 ng·ml^−1^ at 1 h, transiently reaching and slightly exceeding the upper end of the target range. Rapid attainment of a relatively high early exposure was considered important for onset: in the preceding experimental study in dogs, antinociception developed progressively during a 4-h infusion at 5 mg·kg^−1^·h^−1^ and exceeded that of low-dose remifentanil from 60 min onward (10). The loading phase was therefore scheduled so that this interval had elapsed before surgical stimulation began. Following the loading phase, the substantially lower maintenance rate allowed exposure to decline below the operational ceiling. Because the relevant question was whether inter-individual variability could result in sustained over-exposure, the upper tail of the simulated distribution rather than the mean was used for this assessment: the 95th percentile of the combined plasma concentration was approximately 998 ng·ml^−1^ at 1.25 h, 825 ng·ml^−1^ at 2 h, and 539 ng·ml^−1^ at 5 h, indicating that approximately 95% of simulated animals were at or below the ceiling from 1.25 h onward. This front-loaded profile was intended to provide the highest exposure during the intraoperative period, when nociceptive input is greatest, while maintaining continuous but progressively lower exposure throughout the postoperative observation period.

Several limitations of this study warrant consideration. First, the residual analgesic effects of intraoperative remifentanil rescue may have confounded hemodynamic assessments at surgical events subsequent to the first rescue administration in the N/S group; this represents an inherent constraint of rescue analgesia-based intraoperative assessment designs. Remifentanil was selected as the intraoperative rescue analgesic agent due to its rapid onset of action (approximately 1 min) and rapid offset following discontinuation (3–10 min), properties that minimize carry-over analgesic effects between successive surgical events (19). Given the rapid blood–effect site equilibration of remifentanil, peak effect is achieved shortly after administration; accordingly, the subsequent surgical event was initiated after the analgesic effect had peaked but prior to complete offset, to avoid excessive prolongation of surgical time (19, 27). This protocol represents a pragmatic compromise between minimizing residual drug effects and maintaining acceptable operative duration; residual analgesic effects of remifentanil between surgical events therefore cannot be entirely excluded. The limited between-group differences in hemodynamic responses at surgical events other than E2 may partly reflect the confounding influence of repeated remifentanil administrations in the N/S group, which would have blunted subsequent nociceptive responses independently of the study drug. This represents an inherent limitation of rescue analgesia-based intraoperative assessment designs and should be considered when interpreting the LMM results at later surgical events. Second, the canine castration model is associated with mild-to-moderate postoperative pain, which may have reduced the discriminatory sensitivity of the GCMPS-SF across all postoperative time points; validation in surgical models associated with greater nociceptive intensity is therefore warranted (20). Third, this study was conducted exclusively in male Beagle dogs; generalizability to other breeds, body weights, sexes, and surgical models requires further investigation. Fourth, plasma concentrations of opiranserin were not measured, precluding confirmation of the exposure actually achieved and limiting pharmacokinetic–pharmacodynamic characterization. No published pharmacokinetic data exist for opiranserin in dogs; the terminal half-life in healthy human volunteers ranged from 1.51 to 2.13 h across doses of 0.25–8 mg·kg^−1^ (5), but species-specific differences in clearance, distribution, and target receptor pharmacology (GlyT2 and 5-HT2A) remain uncharacterized. Consequently, the regimen used in this study remains investigational. Because a canine therapeutic concentration–response relationship has not been established, it should not be interpreted as an optimized clinical dose, and the optimal dose, dosing interval, and route of administration in dogs remain to be determined through dedicated pharmacokinetic and dose-ranging studies. Fifth, the study lacked a positive analgesic control group; comparison with an established perioperative analgesic would have provided a reference benchmark for evaluating the magnitude of opiranserin's analgesic effect. Sixth, the very small sample size (*n* = 4 per group) and the absence of an *a priori* sample size calculation mean this study was not powered to detect clinically meaningful differences in most outcomes; statistically significant findings should therefore be interpreted as hypothesis-generating rather than confirmatory. Seventh, the omission of opioid premedication and locoregional analgesia deviates from current multimodal analgesic recommendations for canine castration (1) and limits the external validity of these findings. This design was necessary to isolate the analgesic contribution of opiranserin, and animal welfare was safeguarded by baseline meloxicam in all dogs and by predefined intraoperative and postoperative rescue protocols; nevertheless, the results are not directly applicable to routine clinical practice, in which multimodal analgesia would be used. Eighth, the multiplicity of statistical comparisons across numerous primary and secondary outcomes increases the risk of Type I error; Bonferroni correction was applied where appropriate, but this cannot fully mitigate the inflated false-positive risk inherent to an exploratory study with many endpoints.

## Conclusion

In this pilot study, opiranserin was associated with reduced intraoperative rescue analgesia requirements and preliminary evidence of perioperative analgesic benefit in dogs undergoing castration, with lower postoperative pain scores observed at the first hourly postoperative assessment (T1). These findings are hypothesis-generating and require confirmation in larger, adequately powered studies.

## Data Availability

The original data will be made available by the corresponding author upon reasonable request.
